# Missing data in bioarchaeology I: A review of the literature

**DOI:** 10.1002/ajpa.24609

**Published:** 2022-08-23

**Authors:** Amanda Wissler, Kelly E. Blevins, Jane E. Buikstra

**Affiliations:** ^1^ Department of Anthropology University of South Carolina Columbia South Carolina USA; ^2^ Archaeology Department Durham University Durham UK; ^3^ Center for Bioarchaeological Research, School of Human Evolution and Social Change Arizona State University Tempe Arizona USA

**Keywords:** bioarchaeology, literature review, missing data

## Abstract

**Objectives:**

Missing data are a frequent and unavoidable challenge in bioarchaeological research, yet researchers seldom make explicit statements about the bias and inferential limitations that missing data introduce into their studies. There are no guidelines for best practices for the treatment or reporting of missing data. As an initial step in taking stock and exploring approaches to missing data in bioarchaeology, this study reviews bioarchaeological publications to identify methods currently in use for addressing this significant problem.

**Materials and Methods:**

Over 950 bioarchaeology articles (2011–2020) from four major anthropology journals were surveyed, searching for the terms “missing,” “absent,” “unobserv,” “replace,” and “imputat.” The 267 articles so identified were categorized into one of nine bioarchaeological subtopics and scored according to a set of six broad approaches for handling missing data.

**Results:**

Results indicate that bioarchaeologists handle missing data in a variety of ways. Methods such as antimere substitution, listwise deletion and pairwise deletion are widely used. Subject subtopics favor different techniques for handling missing values. Bioarchaeological articles categorized as archaeology, pathology, and trauma used basic missing data approaches, while those such as biodistance and morphology more often employed advanced statistics. Despite the ubiquity of missing data, considerations of how they introduce bias were uncommon and standards for reporting were inconsistent.

**Conclusions:**

These findings highlight areas in which bioarchaeologists can improve techniques for handling and reporting missing data. Greater attention to these shortcomings will increase the statistical rigor of the field.

## 1 INTRODUCTION

Missing data commonly occur in nearly all types of quantitative research, including medicine, ecology, psychology, education, communication, and biology (Altman & Bland, 2007; Dong & Peng, 2013; Enders, 2010; McKnight et al., 2007; van Buuren, 2018). However, most introductory statistics texts do not discuss missing data, their causes, treatment options, or their influence upon the validity of statistical analyses (Allison, 2001; Altman & Bland, 2007). This lack of attention to missing data means that most researchers simply delete cases, individuals, or variables that are missing values with little understanding of how these efforts may introduce bias (Acock, 2005; Enders, 2010; Harel et al., 2008; King et al., 1998). Many scholars may be unaware that alternative options for handling missing data exist (McKnight et al., 2007). Despite an abundance of approaches for handling missing data, they are rarely discussed in most fields and often go unreported in individual studies (Harel et al., 2008; Lang & Little, 2018; Powney et al., 2014; Sylvestre, 2011; Wood et al., 2004). There is an unrecognized taboo against discussing missing data, perhaps due to concerns that a study with missing data was badly designed or poorly executed (van Buuren, 2018). To avoid such censure, authors often gloss‐over areas of their sample with missing values, sometimes eliminating entire variables and sub‐groups behind‐the‐scenes. Seemingly minor details such as pre‐analysis data treatments and excluded samples are often removed due to word limits. Missing data have been described by researchers as a “dirty little secret” (Peugh & Enders, 2004, p. 540) and may be widely regarded as “a nuisance that is best hidden” (Burton & Altman, 2004, p. 6).

Missing data are critical components of data planning, collection, and analysis, and they should be reported and discussed. Including information on excluded samples, pre‐analysis data treatments, and missing values facilitates study transparency and repeatability, particularly for students and early career researchers who may be unfamiliar with the standard protocols. Discussing causes and patterns of missing data in the study sample informs the reader of important biases in recovery, preservation, and curation, which are essential components of a baseline assumption of bioarchaeological research: the study sample is representative of the larger unit being investigated, whether that is a community or regional population. Incorporating and exploring missing data provides a more holistic and less biased understanding of all the data, maximizing a researcher's time, energy, and finances. Clarity in the study design, sample composition, and execution helps the authors, reviewers, and readers evaluate the research, assess the interpretations, and is essential for the study to be included in meta‐analyses (Von Elm et al., 2007).

Missing data have a significant impact on possible statistical tests, such as multivariate analyses that do not allow any missing values (Peng et al., 2007). Multivariate methods incorporate multiple variables in a single test, allowing the researcher to simultaneously control and examine interaction effects, as well as investigate relationships between many variables. Compared to univariate approaches, such methods facilitate a more realistic understanding of how study outcomes are influenced by the interaction of biological, social, and material variables. However, most multivariate statistical methods, such as principal components analysis, discriminant function analysis, or generalized linear models, do not permit missing data—potentially causing researchers to gravitate to simpler analytical methods and neglect complex statistics that could reveal nuanced patterns in bioarchaeological data.

### 1.1 Missing data in bioarchaeology

Missing data are a pervasive challenge in bioarchaeological research. Preservation and recovery factors beginning at the death of the individual and lasting through conservation affect skeletal element preservation and attendant data quality and quantity. Specialized mortuary treatment, secondary burial practices, taphonomy, burial environment, excavation, cleaning, transport, and curation all shape skeletal assemblages (Gordon & Buikstra, 1981; Nawrocki, 1995; Stodder, 2008; Walker et al., 1988). Archeological and historical assemblages are incomplete, fragmentary, and regularly have taphonomic changes that obscure bone surfaces. Skeletons from documented collections are generally more complete, but still suffer from missing elements taken for destructive sampling and the loss of small bones such as those of the hands, feet, sesamoids, and coccygeal elements. In addition to these postmortem biases in skeletal completeness, antemortem events such as tooth loss and wear can exclude elements and individuals from downstream analyses.

Despite missing data being ubiquitous in bioarchaeological research, few scholars have recognized them as a genuine concern or a potential source of bias. Broader discussions in this area have largely been among dental anthropologists as missing data are common in dental tissues. Data may be deemed unobservable due to antemortem and/or postmortem tooth loss, damage, wear, unusual morphology, caries, or calculus deposits. When missing data are not collected, it is assumed they “did not differ in any way from those that were gathered” (Burnett et al., 2013, p. 539), that is, that the data are missing completely at random (MCAR). In practice, however, this may not be the case. For example, numerous dental anthropologists observe that dental attrition can impact nonmetric trait scores (Burnett, 1998, 2016). Burnett et al. (2013) observe that as the severity of tooth wear increases, so does the percentage of crown traits with high degrees of expression. Likewise at high degrees of wear, low grade expressions of crown traits are recorded as not present or as missing data. Stojanowski and Johnson (2015) similarly find that dental attrition may result in trait downgrading. For example, higher degrees of incisor shoveling are more likely to be found on teeth with more extreme wear. Lower expressions of shoveling have been obliterated on highly worn teeth, so only the most extreme shoveling is scorable. As these types of data are used to support sensitive hypotheses about population movement and affinity, the authors' conclusions show how profoundly missing data can affect inferences about the past when not handled properly.

Cirillo (2017) investigates how missing teeth influence data patterns and resulting interpretations. Generally, teeth lost antemortem or postmortem are scored as unobservable when examining oral pathology. This procedure assumes that the cause of missingness is completely random and that teeth missing antemortem do not differ from those missing postmortem. Cirillo demonstrates, however, that teeth lost postmortem are likely to have unhealthy alveolar bone surrounding the crypt, suggesting that even teeth lost postmortem are not missing randomly. She also notes that not all teeth are equally likely to be lost postmortem. Incisors, for example, with their single, straight roots, are more likely to fall out compared to multi‐rooted molars and introduce further bias into the data.

While few researchers routinely evaluate patterns of missingness in their data, some have developed targeted strategies to compensate for missing values. Examining the prevalence of caries in archeological populations, Lukacs (1995) notes that caries frequency will be underestimated when based only on observed teeth, as severe caries will result in tooth loss. Building upon prior work (Hardwick, 1960; Kelley et al., 1991), Lukacs develops a “caries correction factor” used to calculate the true number of caries in an individual when that individual is missing teeth. Auerbach (2011) develops mathematical formulae for estimating vertebral heights, femoral and tibial lengths, and talocalcaneal height when skeletal elements are absent. Auerbach also draws the reader's attention to the importance of handling missing data properly rather than ignoring them, explaining how patterns of missingness in skeletal samples are usually assumed to be missing at random. While not a correction for missing data, Bartelink (2006) proposes a new schema for recording dental data, permitting more nuanced investigation into patterns of missingness. Based on Buikstra and Ubelaker (1994), Bartelink recommends categories into which missing teeth can be categorized. Examples include: “absent, without associated alveolar bone (unknown when it was lost)” (p. 382) or “absent, with the alveolus remodelled or remodelling, antemortem tooth lost” (p. 382). Clear guidelines on how to record and report missing data are lacking in the bioarcheological literature. Further standardization will allow bioarchaeologists to investigate patterns of missingness broadly and clarify problems that missing data introduce.

Scholars in other areas of the social sciences such as psychology and epidemiology have noted a similar lack of protocols for handling and reporting missing data. As a result, they have developed guidelines aimed at improving standards for missing data management (Burton & Altman, 2004; Jeličić et al., 2009; Von Elm et al., 2007; Wilkinson, 1999). For instance, the STROBE (Strengthening the Reporting of Observational Studies in Epidemiology) initiative released a checklist of 22 items intended to increase the rigor of reporting observational studies which includes describing how the sample size was selected and explaining how missing data were handled (Von Elm et al., 2007; p. 1454). A similar effort in bioarchaeology would improve the consistency and precision of future studies.

As an initial step toward increasing the statistical rigor of missing data treatments in bioarchaeology, this paper surveys the state of missing data by examining methods used to handle missing values and considers how missingness is reported in publications. Guided by the results, we address why accounting for missing data is a critical aspect of scientific rigor and provide recommendations for handling and reporting missing data in bioarchaeology. This paper is intended as a companion to Missing Data in Bioarchaeology II (in press), which leverages the results found here to conduct a case study test of missing data methods using bioarchaeological datasets. The objective of this literature review is to determine if there are commonly used methods for handling missing data in bioarchaeology, whether these methods vary by bioarchaeological subtopic, and if there is any variation in methods and treatment over time.

## 2 MATERIALS AND METHODS

**FIGURE 1:**
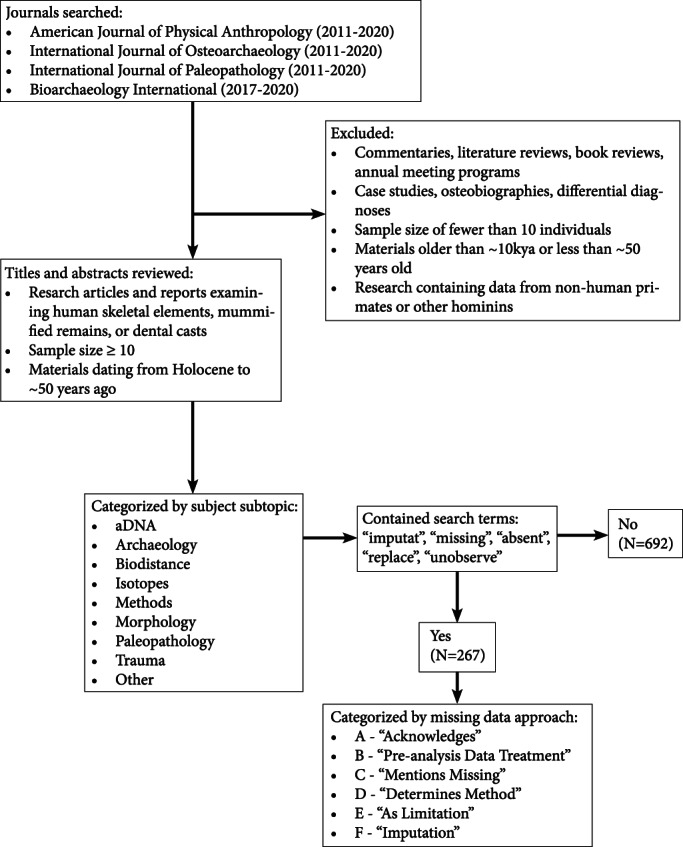
Literature review flow chart

Articles reporting human skeletal elements, mummified remains, or materials derived from human remains (e.g., dental casts) are compiled from the last 10 years from four major anthropology journals: *American Journal of Physical Anthropology* (AJPA, 2011–2020), *Bioarchaeology International* (BI, 2017–2020), *International Journal of Paleopathology* (IJPP, 2011–2020), and *International Journal of Osteoarchaeology* (IJO, 2011–2020). BI began publishing in 2017, therefore only 4 years are included through volume 4 number 1, which was the most recent issue available at the time of the current study. Research articles and reports are included; commentaries, literature reviews, book reviews, and annual meeting programs are excluded. This investigation focuses on population‐level studies, so case studies, osteobiographies, differential diagnoses, and publications reporting a sample size of fewer than 10 individuals are omitted. In choosing to focus upon bioarchaeology, we exclude paleoanthropology and forensic anthropology by including articles studying materials dating to the Holocene (~10 kya) through approximately 50 years ago. The aim is to stay strictly within the purview of bioarchaeology, therefore papers comparing anatomically modern humans to primates or other hominins are also excluded (see Figure 1 for literature review flowchart).

Each article is searched for the following terms: “missing,” “absent,” “imputat,” “replace,” and “unobserv.” Articles that employ any of these words in the context of missing data are compiled for further analysis by the first author. Note that these five terms do not necessarily capture every instance of missing data.

An original goal of this review was to catalog the frequency of specific procedures used during data collection (e.g., antimere substitution) and pre‐analysis data treatments (e.g., listwise deletion, imputation). However, there is a lack of consistency in the language authors use to describe their methods, how they conceptualize their missing data, where in the article missing data are addressed, and whether this information is included in the publication. Literature reviews of missing data in other disciplines have experienced similar difficulties (Klebanoff & Cole, 2008; Lang & Little, 2018; Peugh & Enders, 2004; Powney et al., 2014). As a result, the research aim shifted to explore broader patterns in how bioarchaeological researchers engage with missing data, ranging from data collection/management procedures, theoretical considerations, and discussions of the impact of missing data. How missing data are discussed is therefore categorized according to the following six general missing data approaches (Table 1).

A—“Acknowledges” The authors acknowledge there are values missing from their data. They state, for example, that “unfused epiphyses are commonly missing,” or present summary data and indicate where certain data were unobservable or absent.
B—“Pre‐analysis treatment” The researchers implement procedures during data collection or pre‐analysis data treatment to control for or minimize missing data. Examples include antimere substitution, excluding individuals who do not meet a minimum threshold of completeness, omitting individuals or elements with damage or pathology, or creating an index in which variable categories are collapsed to optimize available data.
C—“Mentions missing” The article discusses missing data generally as a concern—usually in the introduction or in the conclusion—but not directly related to the study sample. For example, “Traditionally, anthropologists have relied on morphological or metric criteria for sex determination, but none of these approaches are 100% accurate, especially when skeletons are incomplete and more sexually dimorphic bones, like the innominate, are absent or are very fragmented” (Garcia, 2012, p. 361).
D—“Determines method” The article mentions the presence of missing data as a reason for choosing a specific statistical method or as an important aspect of the method chosen. For example, numerous studies justify their use of mean measure of divergence as it can handle large amounts of missing data (e.g., Ragsdale & Edgar, 2015).
E—“As limitation” The article cites missing data as a potential limitation for the results and conclusions. The authors discuss how missing data may have reduced the statistical power to detect meaningful differences or how patterns of missingness bias the skeletal sample causing it to be unrepresentative of the original population.
F—“Imputation” The study uses imputation to replace missing data with statistically generated values.

**TABLE 1:** Six missing data approaches

Approach	Explanation
A	“Acknowledges” Acknowledges missing data in the sample For example, “unfused epiphyses were commonly missing”
B	“Pre‐analysis Data Treatment” Uses a collection procedure or pre‐analysis data treatment to control for or minimize missing data For example, substituting right for left; excluding individuals who may be missing certain skeletal elements
C	“Mentions Missing” Mentions missingness in introduction and/or conclusion generally as a concern or limitation
D	“Determines Method” Mentions missing data as a reason for choosing a specific statistical method or as an important aspect of the method chosen
E	“As Limitation” Mentions missing data as a potential limitation of study results For example, renders the sample not entirely representative or limits statistical power
F	“Imputation” Performs imputation or substitution for missing data For example, linear regression, mean replacement

A single article may be assigned to more than one missing data method category. For instance, it is common for articles that performed some type of statistical imputation (Approach F) to first use a method such as antimere substitution (Approach B) to minimize missing data and state that their statistical method allows missing data (Approach D).

Each article is further categorized into one of nine subject subtopics according to the paper's main research question (Table 2). Topics within bioarchaeology have preferred analytical methods, collect unique types of data, and draw from different non‐anthropological fields to inform their methods and theory. Examining how missing data are handled by researchers within these different topics provides insight into broader patterns within the field. Papers on **ancient DNA** explore biological affinity or migration using ancient DNA. **Archaeology** articles use bioarchaeological methods to explore an overall cultural context. Several articles in this category establish the age and sex profiles of a new skeletal assemblage, therefore emphasizing the importance of an archeological site. **Biodistance** articles use metric or nonmetric traits to examine biological affinity and migration. **Isotopes** articles use isotopes or trace elements from skeletons or preserved tissues to examine diet, migration, and past lifeways. **Methods** articles have the goal of creating or testing a method such as age estimation or statistical analysis; they may employ morphology or musculoskeletal markers but the focus of the paper is on the method. Stojanowski and Hubbard (2017) evaluate “what variables and methods best identify known relatives within [a] sample” (p. 814) in biological distance analyses. Since the goal of this paper is to inform and refine biodistance methodology, this paper is placed in “methods” rather than “biodistance.” Articles categorized as **morphology** include studies of tooth shape, stature, and limb and cranial shape (when not used for biodistance studies). **Pathology** articles include those studying health and disease, paleoepidemiology, musculoskeletal markers, dental wear, and cranial and dental modification. **Trauma** studies explore skeletal trauma and past violence. Finally, articles categorized as **other** could not be described as belonging to any of the other eight subtopics.

**TABLE 2:** Nine bioarchaeological subtopics

Category	Description
aDNA	Ancient human DNA to examine migration, biological affinity Ancient pathogen DNA
Archaeology	Using bioarchaeological methods to explore an overall cultural context, or lifeway Performing basic osteological methods to establish a context
Biodistance	Using metric or nonmetric data to examine biological affinity
Isotopes	Using isotopes from skeletal elements to examine diet, migration, lifeways Trace element analysis
Methods	Creating or testing a method (e.g., aging, sexing, statistics, and skeletal index) May use morphology or musculoskeletal markers but the focus of the paper is on the method Investigations of taphonomy
Morphology	Stature (when not in a framework of poor health) Limb and cranial shape (when not used for biological distance) Tooth shape and growth Trabecular architecture
Pathology	Health and disease, paleoepidemiology, Musculoskeletal markers, auditory exostoses Cranial and dental modification, dental wear
Trauma	Skeletal trauma, warfare, violence
Other	Blood type, tooth pulp volume, phytoliths in calculus

## 3 RESULTS

A total of 959 articles meet the criteria for inclusion. Of these, 267 (27.8%) mention missing data using one of the five search terms. A total of 141 are from AJPA, 92 from IJO, 24 from IJPP, and 10 from BI. Eight of the 267 articles could not be meaningfully categorized into a single subject and are thus placed into two categories and double counted. For example, Redfern et al. (2017) examine the association between multiple skeletal trauma and health status; it is therefore placed in both the trauma and pathology categories. The other 692 articles have study designs that discuss missingness in terms other than the five selected, do not have missing data, or do not disclose the presence of missing data. The complete Excel spreadsheet is available under Supporting Information as well as under the first author's GitHub Repository (Wissler, 2022).

**FIGURE 2:**
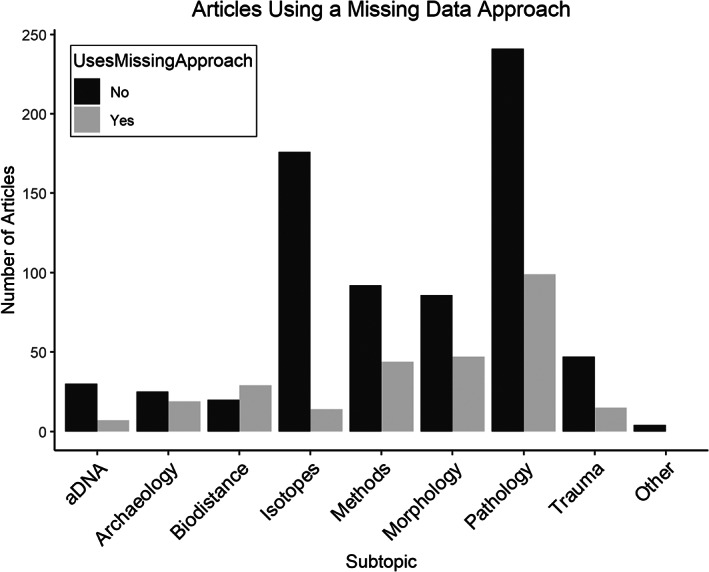
Barplot showing number of articles using a missing data approach by subtopic

Figure 2 shows the number of articles using a missing data approach by subtopic. Overall, only 27.7% of all the articles surveyed engage with missing data. Most subtopics have far more articles that do not use a missing data approach except for biodistance with 59.2% (29 Yes, 20 No) of the articles mentioning missing data. Within archaeology, 43.2% of the articles discuss missing data (19 Yes, 25 No). Methods and morphology have similar results, 32.4% of methods articles and 35.3% of morphology articles mention missing data. A total of 29.1% of pathology articles and only 18.9% of aDNA articles use a missing data approach. Missing data management appears to be particularly uncommon among isotopes publications as only 7.4% (14 Yes, 176 No) mention missing data using one of the five terms. The percentage of articles using a missing data approach varies by journal. *International Journal of Osteoarchaeology* has 43.0% of their articles mention missing data, *Bioarchaeology International* has 30.3%, *American Journal of Physical Anthropology* 25.6% and *International Journal of Paleopathology* 24.2%. A figure with these results is available under Supporting Information.

The following results focus on the 275 articles (which includes the eight double‐counted) that are identified using the five missing data terms. Overall, the most common missing data approach found is B “Pre‐analysis data treatment” in which 132 articles (48.0%) employ a technique during data collection or data cleaning to limit missing data (Table 3). Note that due to double counting eight articles and because a single article may be tallied under more than one missing data approach category the column sums in Table 3 will not add up to 267 or 275. The second most common missing data approach is A “Acknowledges” (*n* = 114, 41.5%), which indicates the presence of missing data in the study. Only 25 articles employ missing data approach C “Mentions missing.” Few articles use missing data approaches D “Determines method” (*n* = 23) or E “As limitation” (*n* = 15). A total of 43 articles use missing data approach F “Imputation.” Results of a Kruskall‐Wallis test show that there is a significant difference in the mean number of articles in each missing data approach (*p*‐value = 0.033). Table 3 presents the number of articles per bioarchaeological subject topic. The majority are in pathology (*n* = 118), followed by morphology (*n* = 66) and methods (*n* = 60) while the fewest are from isotopes (*n* = 15), trauma (*n* = 15), ancient DNA (*n* = 9), and other (*n* = 0).

**TABLE 3:** Summary of literature review results

	A	B	C	D	E	F	Total
aDNA	4	2	2	0	0	1	9
Archaeology	15	4	2	0	1	0	22
Biodistance	1	19	6	6	0	15	47
Isotopes	11	2	0	1	0	1	15
Methods	14	22	5	6	9	4	60
Morphology	16	30	0	2	1	15	64
Pathology	46	48	6	8	3	7	118
Trauma	7	5	2	0	1	0	15
Other	0	0	0	0	0	0	0
Total	114	132	23	23	15	43	

**FIGURE 3:**
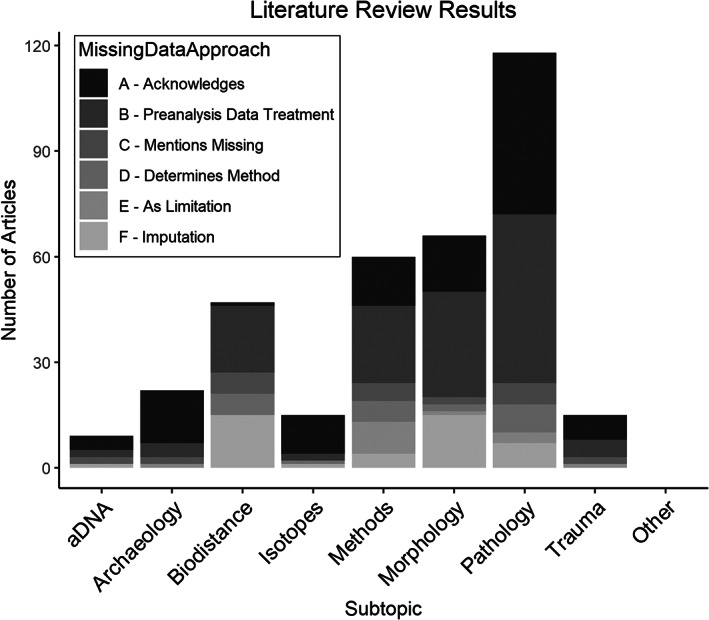
Barplot showing counts of missing data approach by subtopic

Figure 3 shows the percentage of each missing data approach by subject topic (a color version is available under Supporting Information). Given that IJPP and BI focus heavily on skeletal pathology it is unsurprising that there are so many articles in this subtopic. The vast majority (80%) of pathology articles that address missing data do so using approaches A or B. Despite the large number of pathology articles indicating that there are missing data in their samples or discussing missing data in relation to their collection procedures, only 5.5% mention missing data as a potential problem or limitation for their results (Approach E). Morphology contains the second greatest number of articles using a missing data approach (*n* = 66). Not quite half employ missing data approach B “Pre‐analysis data treatment.” Morphology also has the second largest percentage (23%) of articles employing missing data approach F “Imputation.” Comparatively few morphology articles, however, discuss missing data as a potential concern or a limitation for their results (Approach E). A total of 60 articles are categorized as methods, most of which employed missing data approaches A and B. Methods papers also have a high percentage of articles (10%) that consider the ability to handle missing data as an important aspect of their statistical methods selection (Approach D). A total of 47 biodistance articles use a missing data approach, 40% of which use missing data approach B “Pre‐analysis data treatment.” Compared to the other subjects, a greater proportion of the biodistance articles use category D “Determines method” and F “Imputation.” Trauma is among the least common subject topic found in the journals surveyed (*n* = 15) and displays little diversity in the approaches to missing data, as 47% use approach A. Only 15 articles that employ a missing data approach are categorized as “isotopes,” 11 of which acknowledge missing data (Approach A). One isotopes article (Allen et al., 2020) uses imputation, but it is also categorized as biodistance. Finally, only nine articles are assigned to the aDNA subject topic, most of which detail the presence of missing data (Approach A).

**FIGURE 4:**
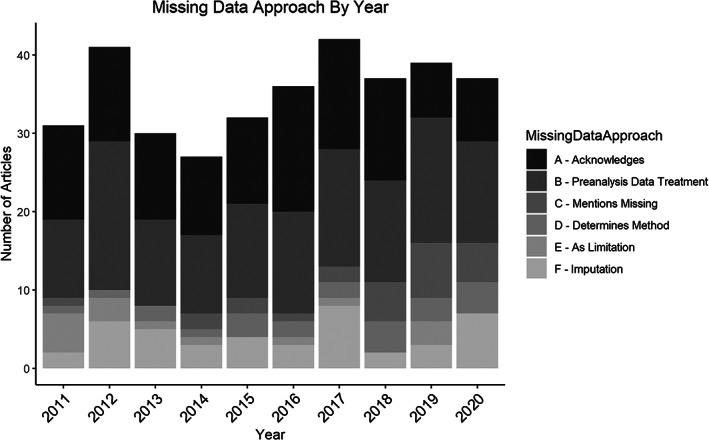
Trends in the usage of missing data approaches over time

Figure 4 shows how patterns in missing data approaches vary over time (a color version is available under Supporting Information). The number of articles in each missingness category remains relatively constant over the past 10 years—indicating very little temporal change despite improvements in statistical software and computing power in the last decade. There is a slight increase in the number of articles that discuss missing data as a limiting factor for their results and interpretations (Approach C). Finally, only four articles (Falys & Prangle, 2015; Luna, 2019; Niinimäki, 2012; Niinimäki & Baiges Sotos, 2013) state that there are no missing data in their sample or that missing data treatments are unnecessary. It is possible that many of the 692 surveyed articles similarly have no missing data but did not mention it in the text.

## 4 DISCUSSION AND CONCLUSIONS

This literature review explores how bioarchaeologists handle missing data by reviewing published articles from the last 10 years from four major journals. Of the 275 articles that use a missing data approach, 132 (48.2%) employ approach B “Pre‐analysis data treatments.” Pre‐analysis data treatments include antimere substitution, deleting individuals with missing data, excluding individuals or elements who did not meet a minimum threshold of completeness, or excluding pathological individuals. The ubiquity of this approach reveals that these are the foundational procedures for handling missing data in bioarchaeology. Indeed, substituting the right element when the left is unavailable is established in *Standards for Data Collection from Human Skeletal Remains* (Buikstra & Ubelaker, 1994) for cranial, postcranial, and dental measurements. Deletion methods are the simplest for dealing with missing data, however, they rely on the most conservative statistical assumptions: less than 5% of the data can be missing and they must be MCAR (Graham, 2009; Little & Rubin, 2020). When patterns of missing data do not meet these criteria, deleting missing variables or individuals can skew the results, presenting an incomplete and biased outcome (Little & Rubin, 2020; McKnight et al., 2007). Bioarchaeological data are likely not MCAR and may therefore fail the requirements for pairwise and listwise deletion (Burnett et al., 2013; Stojanowski & Johnson, 2015). Smaller, lighter, and more fragile bones such as those belonging to children, older adults, or individuals with severe pathological conditions may be less likely to preserve over time or be recovered during excavation (Bello et al., 2006; Gordon & Buikstra, 1981; Holt & Benfer, 2000; Mays, 1992; Stojanowski et al., 2002; Walker et al., 1988). Such biases are an inherent and yet unknowable part of bioarchaeological data. Furthermore, these findings suggest that missing data are anticipated and planned for in bioarchaeological studies despite little discussion of missing data in the field. Few authors explicitly consider missing data when selecting statistical analyses (Approach D), indicating that analytical techniques are infrequently dictated by missing data. Examining the impact of missing data (Approach E) is rare as are wider discussions of the statistical and interpretive limitations imposed by missing data (Approaches C and D)—particularly given the number of articles explicitly identifying missing values in their skeletal datasets.

Each bioarchaeological subject subtopic has its own preferred techniques for conceptualizing and handling missing data. Pathology and trauma articles tend to focus on highly contextualized patterns of pathology and trauma, and their data are more likely to be counts of particular lesions or injuries. General descriptive statistics and univariate analyses may be appropriate in these cases and more sophisticated techniques to handle missing data viewed as unnecessary in these studies. Authors of biodistance and morphology articles—areas that tend to be the most statistically advanced in bioarchaeology—more often use statistical methods that allow missing data and are cognizant of analytical methods that can be biased by missingness. This may be because multivariate statistics, such as those used in biodistance or morphological analyses, typically do not permit missing data—causing scholars in these areas to manage their missing data on a statistically more sophisticated level than other topics.

Our results indicate it is not standard practice for bioarchaeologists to critically examine patterns of missingness in their data, either during study design or in publication. Of the over 950 articles included in this study, only 27.8% mention missing data. Furthermore, the paucity of articles with the stated goal of managing missing data (e.g., Auerbach, 2011) suggests that bioarchaeology is not critically engaging with missing data—a concern given the ubiquity of missing data in the field. This lack of engagement indicates that researchers do not understand how missing data may bias statistical analyses and ensuing results and conclusions. For example, focusing only on complete datasets privileges certain contexts with better preservation potentially rendering their conclusions unrepresentative of broader regional trends (Auerbach, 2011; Holt & Benfer, 2000). Why there is such a mismatch between the obvious presence of missing data in bioarchaeology and the number of practitioners using techniques to manage missing data is unclear and warrants further inquiry. We speculate it may partially be due to an unwillingness to reveal the amount of data missing from a project as it may appear to undermine a study's strength.

The management of missing data in bioarchaeology has important implications for the scientific rigor of the field. Missing data can substantially decrease sample sizes, limiting the power to detect meaningful differences between groups (Graham, 2009; Kang, 2013; McKnight et al., 2007; Peng et al., 2007). Compounding the problem, most bioarchaeological studies do not perform power analyses so it is unclear whether those with small sample sizes can produce meaningful results. Failure to disclose missing data can create uncertainty in a research article related to differential sample sizes used for separate univariate analyses. If pairwise deletion is performed but not described, the number of individuals listed in one section may not match the number presented in another.

Scholars in other fields have recognized similar systemic inconsistencies in missing data reporting and therefore have created guidelines to improve the rigor of research design and publishing in their respective areas (Akl et al., 2015; Burton & Altman, 2004; Jeličić et al., 2009; Wilkinson, 1999). Following their example, we propose several recommendations to increase bioarchaeological engagement with missing data and transparency in study design. (1) Bioarchaeologists should publish detailed descriptions of data collection procedures, explaining how individuals were selected for inclusion. (2) Researchers should document specific causes of missing data (e.g., is the tooth missing, broken, worn, unerupted, etc.) rather than only recording “NA.” (3) Publications should include any pre‐analysis data treatments or data cleaning, as well as justifications for these decisions. (4) Authors should disclose when missing data are present—or if there are no missing data—and provide exact numbers of individuals and variables excluded for each analysis. (5) Discussion sections should describe how missing data impact sample representativeness and research findings. (6) When appropriate, implement Little's MCAR test (Little, 1988) to reveal patterns in missing data and indicate when missing data may be problematic (see Burnett et al., 2013; Stojanowski & Johnson, 2015). Numerous statistical tutorials and packages for this test exist for R, SPSS, and Stata. Given word limits for publications, this information could be included as Supplemental Information.

This study has several limitations. Four of the most well‐known journals in bioarchaeology were chosen for analysis; it is possible that papers engaging in critical discussions of missing data theory and procedures to handle missing data may be published in methods‐oriented journals or so‐called gray literature including dissertations and theses. As mentioned above, articles included in our analysis are identified using the five keywords. Those discussing missing data without using these keywords are not included; our results may therefore underestimate certain types of missing data approaches. We provide an overview of missing data in bioarchaeology only and do not provide comparative data from other areas. Further research of missing data management in other fields in anthropology such as archaeology or evolutionary anthropology would provide a greater understanding of how anthropologists as a whole handle missing data and provide guidance for bioarchaeologists.

Overall, our results suggest that bioarchaeology lacks a strong foundation in missing data management. The large percentage of articles not addressing missing data indicates that researchers do not fully understand the implications of missing data which impact sample representativeness and the validity of statistical tests. Small steps such as clearly reporting pre‐analysis data treatments and patterns of missingness in publications, discussing the biases and limitations missing data presents, and exploring alternative methods such as imputation will improve the statistical rigor of our analyses.

## AUTHOR CONTRIBUTIONS

**Amanda Wissler:** Conceptualization (lead); data curation (lead); formal analysis (lead); funding acquisition (lead); investigation (lead); methodology (lead); project administration (equal); resources (lead); software (lead); supervision (equal); validation (lead); visualization (lead); writing – original draft (lead); writing – review and editing (lead). **Kelly E. Blevins:** Validation (equal); writing – original draft (supporting); writing – review and editing (equal). **Jane E. Buikstra:** Supervision (equal); writing – review and editing (supporting).

## CONFLICT OF INTEREST

The authors declare no potential conflict of interest.

## Supporting information


**Appendix S1** Supporting Information

## Data Availability

The complete Excel spreadsheet is available under Supporting Information as well as under the first author's GitHub Repository (Wissler, 2022).
