# Supplementary figures and images for: Missing data in bioarchaeology I: A review of the literature
Source: Am J Biol Anthropol. 2022 Aug 23;179(3):339–48. doi: 10.1002/ajpa.24609 (PMC9804890; doi:10.1002/ajpa.24609)

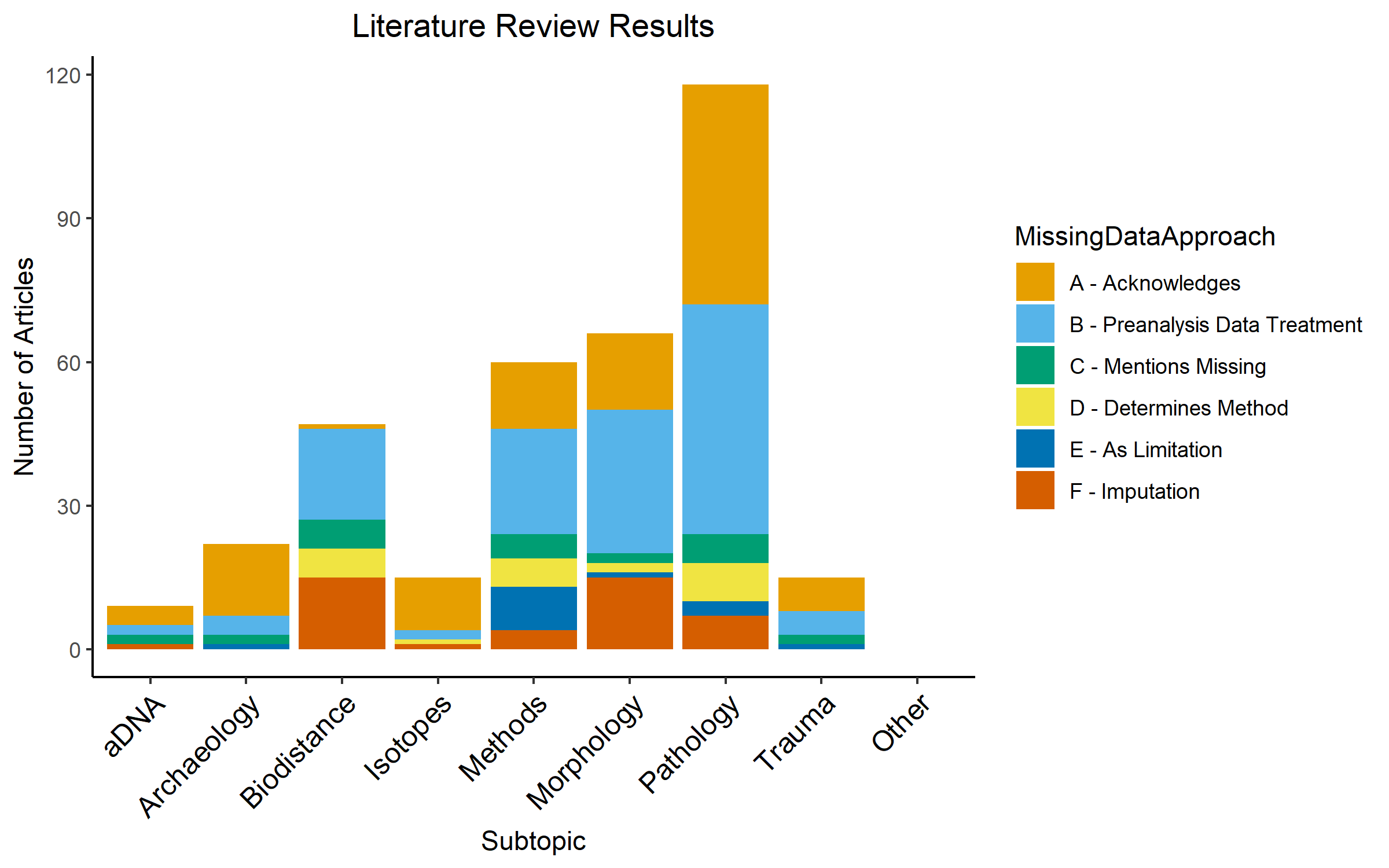

Supplement: Supplementary file 1 — Appendix S1 Supporting Information [file AJPA-179-339-s001.zip › AJPA_24609_Figure_3_Color_SuppInfo.tif]

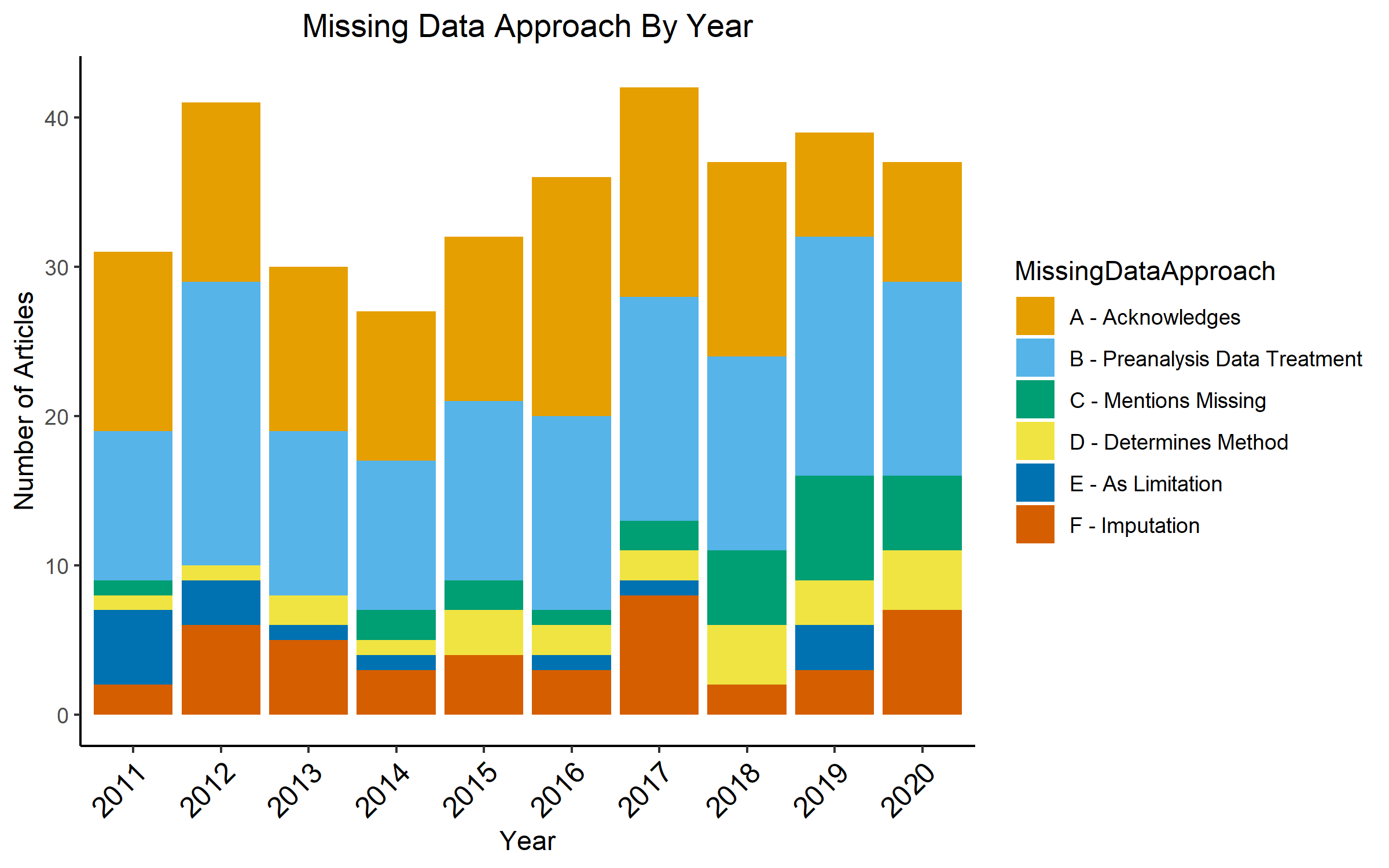

Supplement: Supplementary file 1 — Appendix S1 Supporting Information [file AJPA-179-339-s001.zip › AJPA_24609_Figure_4_Color_SuppInfo.tif]

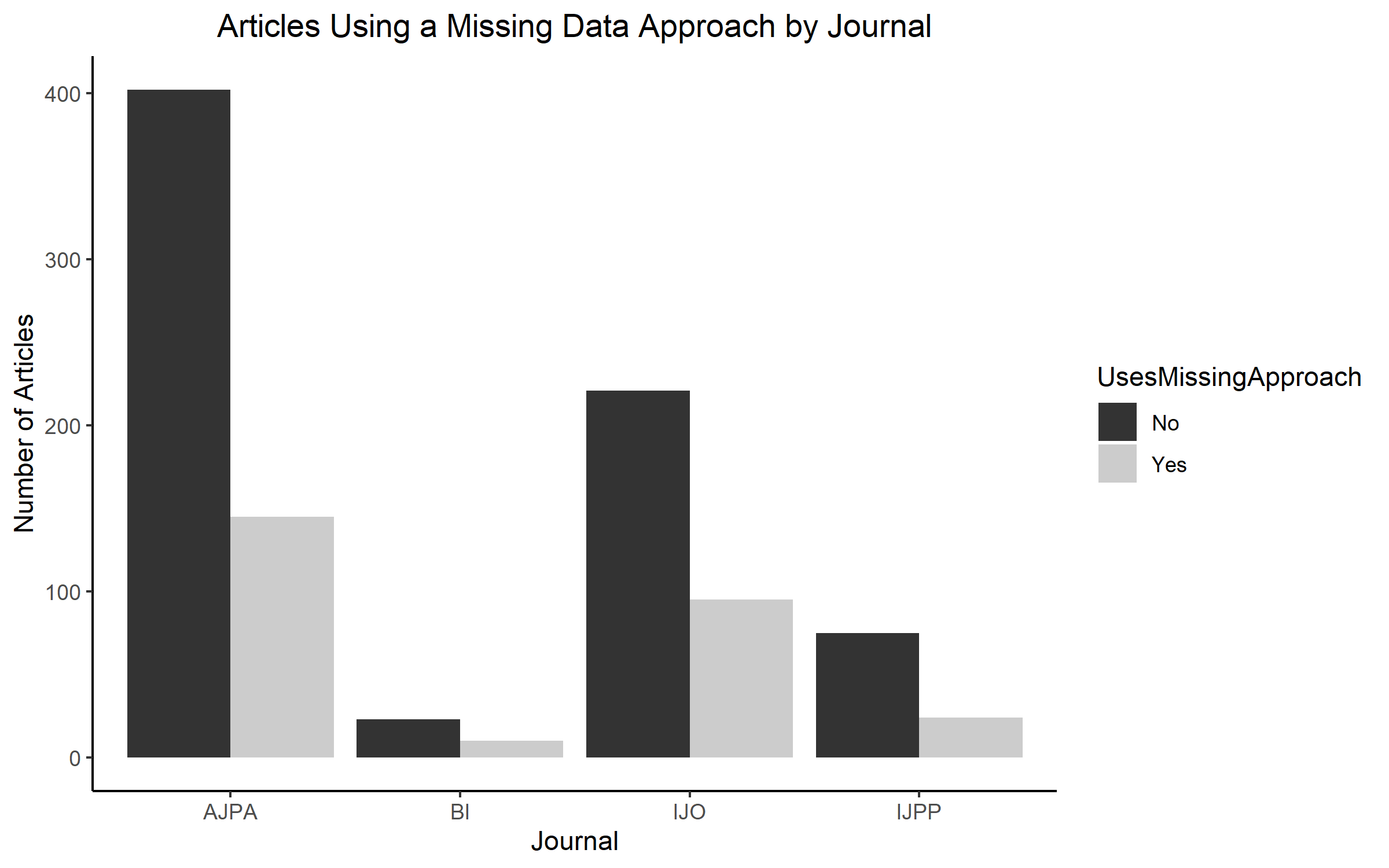

Supplement: Supplementary file 1 — Appendix S1 Supporting Information [file AJPA-179-339-s001.zip › AJPA_24609_Figure_5_SuppInfo.tif]
